# The Incorporation of Sulfonated PAF Enhances the Proton Conductivity of Nafion Membranes at High Temperatures

**DOI:** 10.3390/polym16152208

**Published:** 2024-08-02

**Authors:** Kun Cai, Jinzhu Yu, Wenjun Tan, Cong Gao, Zili Zhao, Suxin Yuan, Jinghui Cheng, Yajie Yang, Ye Yuan

**Affiliations:** 1 Key Laboratory of Micro-Nano Materials for Energy Storage and Conversion of Henan Province, Institute of Surface Micro and Nano Materials, College of Chemical and Materials Engineering, Xuchang University, Xuchang 461000, China; yjz020914@outlook.com (J.Y.); tanwj@dhu.edu.cn (W.T.); galigiaogiaoBBB@163.com (C.G.); y1061582635@163.com (S.Y.); jinghui871016@163.com (J.C.); 2 XuJue Electric Co., Ltd., Xuchang 461000, China; 13513748576@163.com; 3 Key Laboratory of Automobile Materials of Ministry of Education, School of Materials Science and Engineering, Jilin University, Changchun 130022, China; 4 Key Laboratory of Polyoxometalate and Reticular Material Chemistry of Ministry of Education, Northeast Normal University, Changchun 130024, China; yuany101@nenu.edu.cn

**Keywords:** Nafion, composite membrane, proton conduction, PAFs

## Abstract

Nafion membranes are widely used as proton exchange membranes, but their proton conductivity deteriorates in high-temperature environments due to the loss of water molecules. To address this challenge, we propose the utilization of porous aromatic frameworks (PAFs) as a potential solution. PAFs exhibit remarkable characteristics, such as a high specific surface area and porosity, and their porous channels can be post-synthesized. Here, a novel approach was employed to synthesize a PAF material, designated as PAF-45D, which exhibits a specific surface area of 1571.9 m^2^·g^−1^ and possesses the added benefits of facile synthesis and a low cost. Subsequently, sulfonation treatment was applied to PAF-45D in order to introduce sulfonic acid groups into its pores, resulting in the formation of PAF-45DS. The successful incorporation of sulfonic groups was confirmed through TG, IR, and EDS analyses. Furthermore, a novel Nafion composite membrane was prepared by incorporating PAF-45DS. The Nyquist plot of the composite membranes demonstrates that the sulfonated PAF-45DS material can enhance the proton conductivity of Nafion membranes at high temperatures. Specifically, under identical film formation conditions, doping with a 4% mass fraction of PAF-45DS, the conductivity of the Nafion composite membrane increased remarkably from 2.25 × 10^−3^ S·cm^−1^ to 5.67 × 10^−3^ S·cm^−1^, nearly 2.5 times higher. Such promising and cost-effective materials could be envisioned for application in the field of Nafion composite membranes.

## 1. Introduction

Proton exchange membrane fuel cells (PEMFCs) are widely acknowledged for their high energy conversion efficiency and environmental friendliness [1]. Proton exchange membranes (PEMs), a crucial component of fuel cells, facilitate the migration and transport of protons while simultaneously serving as a barrier for gas reactants. Nafion, developed by DuPont, is a perfluorosulfonic acid (PFSA) membrane. It is the most commercialized proton exchange membrane and exhibits high proton conductivity under moderate temperature conditions [2]. However, Nafion membranes exhibit certain drawbacks, such as slightly elevated methanol permeability and reduced proton conductivity due to the evaporation of water molecules responsible for proton conduction at high temperatures [3,4,5]. Consequently, numerous mixed matrix membranes incorporating Nafion have been attracting attention. In recent years, novel porous materials such as metal organic frameworks (MOFs) [6,7,8,9,10] and covalent organic frameworks (COFs) [11,12,13,14] have been employed as fillers. As members of the porous material family, porous aromatic frameworks (PAFs) can also serve as fillers due to their exceptional resistance to acid and heat. However, to the best of our knowledge, there are limited reports on the synthesis of PAF-doped Nafion composite membranes.

PAFs, constructed via robust C–C covalent bonds, exhibit numerous exceptional properties, including a high specific surface area and porosity, and have been extensively studied in gas storage or separation [15,16,17], catalysis [18,19,20], and other domains [21,22,23,24,25,26,27]. Furthermore, they demonstrate notable thermal and chemical stability [16], capable of enduring post-synthetic modification under harsh conditions, such as treatment with chlorosulfonic acid [28,29,30,31]. Due to these advantageous characteristics, this paper proposes using PAF materials as novel fillers to address the dehydration issue of Nafion membranes at high temperatures and low humidity. The pores of PAFs have the ability to adsorb water molecules and preserve the proton transport channel. By sulfonating the framework, the hydrophilicity of the pores is enhanced, which also facilitates the substantial supply of protons. However, some PAFs still have certain disadvantages, such as difficulties in monomer synthesis and the need for expensive catalysts. Therefore, PAF-45 was selected.

PAF-45, a member of the PAF family [32], possesses the added benefits of facile synthesis and a low cost. Among its raw materials, the biphenyl monomer and the aluminum chloride catalyst are comparatively economical. This attribute is particularly significant for Nafion membrane fillers as Nafion itself is highly expensive. Therefore, PAF-45 was selected as the dopant. According to the literature [32,33], a novel approach was employed in synthesizing PAF-45 by substituting trichloromethane with dichloromethane, resulting in a material with a larger surface area (1571.9 m^2^·g^−1^), designated as PAF-45D (D = dichloromethane). Then, PAF-45D was treated with chlorosulfonic acid to introduce sulfonic acid groups into its pores, resulting in the product marked PAF-45DS [29,34]. This sulfonation process effectively enhances the hydrophilicity of the PAF-45D pores. These modifications are expected to enhance the conductivity of Nafion films at elevated temperatures. Subsequently, the impact of doping PAF-45DS with varying mass fractions on the proton conductivity of Nafion composite membranes was primarily investigated. Under identical film formation conditions, the conductivity of the Nafion composite membrane increased significantly from 2.25 × 10^−3^ S·cm^−1^ to 5.67 × 10^−3^ S·cm^−1^ at 80 °C ~78% RH (relative humidity), nearly 2.5 times, when only doping with a 4% mass fraction of PAF-45DS. This represents a substantial enhancement in membrane performance.

## 2. Materials and Methods

All commercial chemical reagents were used without further purification. The main reagents were biphenyl (AR, Aladdin Reagents, Shanghai, China), AlCl_3_ (AR, Aladdin Reagents, Shanghai, China), Nafion (D520, DuPont, Wilmington, DE, USA), chlorosulfonic acid (AR, Aladdin Reagents, Shanghai, China), and dichloromethane (AR, Sinopharm Group SCR Reagents, Beijing, China). Thermogravimetric analyses (TGA) were performed under an atmosphere with a heating rate of 10 °C min^−1^ using a STA 409 PC from 30 °C to 800 °C. Before testing, the sample was cleaned with acid and water. Powder X-ray-diffraction (XRD) analyses were performed on a Bruker D8 Advance with Cu Kα (λ = 1.5418 Å) at 40 kV, 40 mA. Fourier-transform infrared spectra (FT-IR) were obtained on a Thermo Nicolet Nexus 470 FT-IR (Thermo Fisher Scientific, Waltham, MA, USA) spectrometer in the 4000–400 cm^−1^ range using KBr pellets. The N_2_ gas sorption measurements were acquired on a BELSORP-max (MicrotracBEL Japan Inc., Osaka, Japan) surface area analyzer at 77 K. The water uptake was analyzed on a JWGB Instrument JW-ZQ100 (JWGB Instrument, Beijing, China) at 298 K. The SEM and EDS pictures were taken on a Nova NanoSEM 450 (FEI Company, Hillsboro, OR, USA).

### 2.1. Synthesis of PAF-45D

In the experiment, the catalyst was activated first. Anhydrous aluminum chloride (1 g) and dichloromethane (20 mL) were added to a 100 mL round-bottom flask, and the catalyst was stirred at 65 °C for 3 h. After activation, 200 mg of biphenyl was dissolved in 20 mL of dichloromethane and poured into the round-bottom flask. After the reaction system was stirred in an oil bath at 65 °C for 24 h, the power was turned off, and the crude product was obtained after the temperature of the reaction system had dropped to room temperature. Ethanol (50 mL) was added to the product, which was filtered with a pump. To remove the raw materials and catalysts, the resulting material was added to the beaker with 50 mL 2 mol·L^−1^ HCl and stirred for 3 h, followed by ultrasonication for 30 min, and then filtrated. Subsequently, distilled water and ethanol were used successively. The agitation, ultrasonication, and filtration process was repeated. The cleaning procedure was iterated three times. The final product was placed in an oven at 80 °C to dry, and it was named PAF-45D.

### 2.2. Synthesis of PAF-45DS

PAF-45D (250 mg) and dichloromethane (20 mL) were added to a 100 mL flask, which was filled with N_2_ gas and cooled in an ice-water bath. Chlorosulfonic acid (4.0 mL) was slowly dropped into the flask. The mixture was stirred at room temperature for three days; then, the product was poured into 50 mL ice water under stirring conditions. Then, ethanol (50 mL) was added to the water; it was filtered with a pump and then washed with distilled water and ethanol three times successively. The treatment method was the same as above. The final product was placed in an oven at 80 °C to dry, and it was named PAF-45DS.

### 2.3. Preparation of Nafion Composite Membrane

Nafion D520 is available for commercial use and its solvent is an alcohol–water mixture. However, it is necessary to convert the solvent into a DMF solution in order to achieve the better dispersion of PAF. A certain amount of Nafion D520 was placed in a glass culture dish and positioned in an 80 °C oven for drying. Then, the dry Nafion film was cut into pieces and a certain amount of DMF was added to prepare a 5% (wt.%) Nafion–DMF solution. The conventional solution casting technique was carried out to prepare the composite membranes. Different masses of PAF-45DS (0 wt.%, 2 wt.%, 4 wt.%, 6 wt.%, 8 wt.%, and 10 wt.%) were added to the Nafion–DMF solution, which was then subjected to ultrasonic dispersion for half an hour to obtain the film-forming liquid. Then, it was dropped onto a flat Teflon surface and left at 80 °C for 12 h until the Nafion composite films were obtained.

### 2.4. Proton Conductivity

The proton conductivity of the membranes was measured via the AC impedance spectroscopy technique with a quasi-four-probe method on a Solartron ModuLab XM (AMETEK SI, Berwyn, PA, USA). A frequency from 1 MHz to 1 Hz, 5 mV AC perturbations, and a 0.0 V DC rest voltage were used during the test. Saturated solutions of KCl were used to act as a humidity source with about 78% relative humidity (~78%RH) at 80 °C. The solution was placed into a sealed bottle for 3 days to ensure that the air reached an equilibrium state. The testing temperature of the impedance studies was generated by water vapor at 80 °C. The Nafion composite films were cut to round pieces with a diameter of 3 mm, and the thickness of the films was tested with a micrometer caliper. Prior to the measurement, the samples were placed in a bottle at the required temperature of 80 °C for a certain time. The conductivity (*σ*, S cm^−1^) was calculated using the relation *σ* = *L*/(*RA*), where *R* (Ω) is the sample resistance estimated by the extrapolation of the high-frequency arc crossing to the axis [35]. *L* (cm) is the thickness and *A* (cm^2^) is the face area.

## 3. Results and Dicussion

### 3.1. X-ray Diffraction Analysis

**Figure 1:**
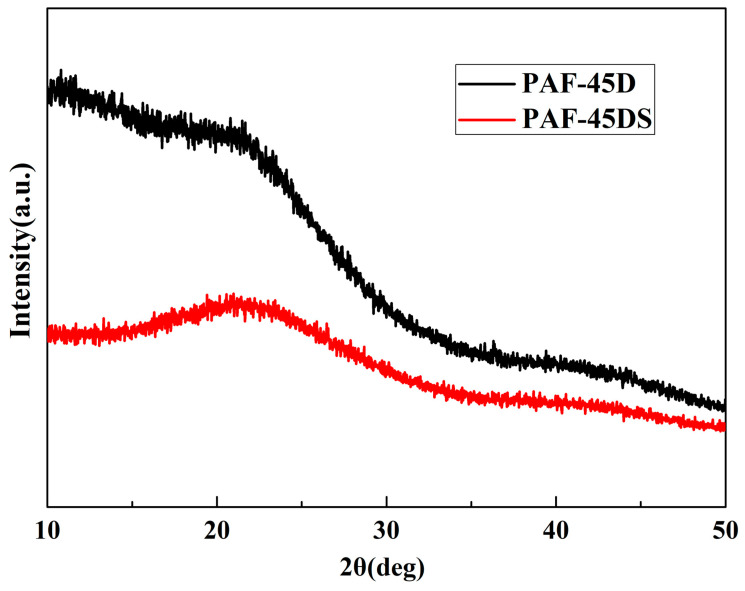
XRD patterns of the PAF powder.

The X-ray diffraction patterns of the samples and the Nafion composite films with different doping amounts were tested. Both the powder sample and the membrane sample were tested on a glass plate with grooves. It can be seen from Figure 1 that the peaks of PAF-45D and PAF-45DS are basically the same, and it shows typical amorphous polymer peaks. This is consistent with the characteristics of the previously reported material [32]. There are no weak peaks for the PAF-45D material, which suggests that the catalyst AlCl_3_ was basically removed. The curve of PAF-45DS is an amorphous material curve, indicating that no impurities were introduced during the sulfonation.

**Figure 2:**
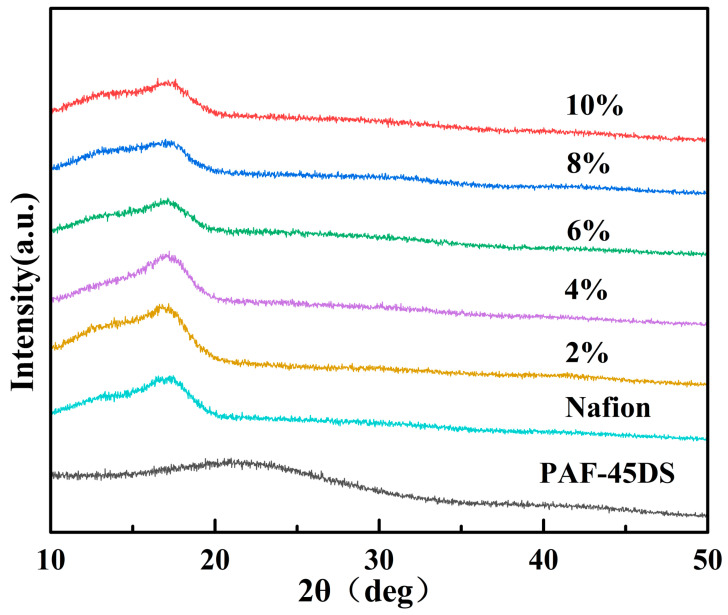
XRD patterns of Nafion composite films with various content of PAF-45DS.

Figure 2 illustrates the XRD patterns of the Nafion composite films doped with different mass fractions of PAF-45DS. It can be observed that as the mass fraction of PAF-45DS increases, the peak position of the Nafion composite films shifts to the right and approaches the amorphous peak of PAF-45DS, indicating the gradual intensification of the amorphous nature of the Nafion composite film.

### 3.2. TGA and IR

**Figure 3:**
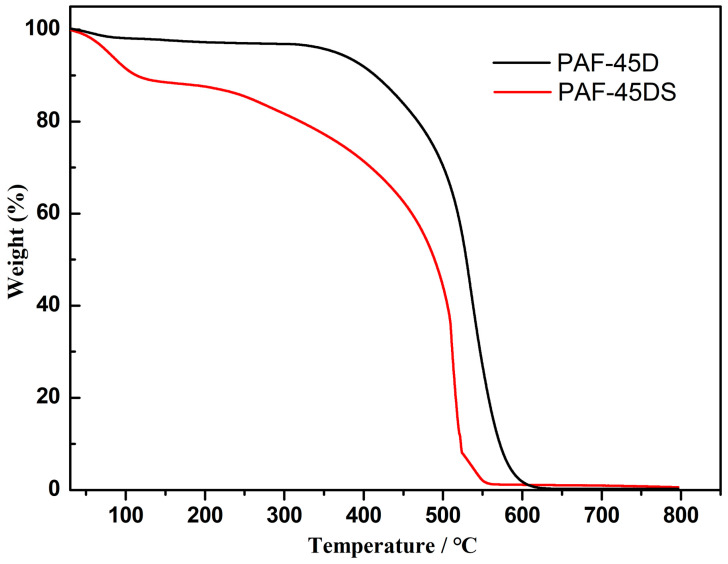
TGA thermograms of PAF-45D and PAF-45DS.

The thermal stability of PAF-45D and PAF-45DS was investigated through thermogravimetric analysis (TGA) experiments. The TG curve was recorded in an air atmosphere from 35 to 800 °C (Figure 3). PAF-45D has less weight loss before 100 °C. After a platform, another weight loss is observed starting from ~350 °C, and the mass begins to drop sharply, which is attributed to the decomposition of the polymer frameworks. A weight loss of 11.2% is observed from 35 to 126 °C in the curve of PAF-45DS, which is attributed to the release of adsorbed water in the pores. After a brief plateau, the mass begins to decrease continually from 200 °C, indicating the collapse of the skeleton. The TGA demonstrates that the introduction of sulfonic acid groups reduces the thermal stability of the material. More importantly, sulfonation enhances the water absorption capacity of PAF-45D.

To determine whether the sulfonic acid group was introduced, Fourier transform infrared spectroscopy (FT-IR) was performed on PAF-45D and PAF-45DS. As shown in Figure S1, the band associated with the S–O vibration is detected at 1176 cm^−1^ in the spectrum of PAF-45DS, which is similar to the literature reports [29]. The range 1660~1373 cm^−1^ in the spectrum denotes the C=C stretching vibrations for the benzene ring. The spectroscopy confirms the existence of alkyl groups in the structure by C-H stretching vibrations near 2929 cm^−1^. This is consistent with the literature report [32,33]. Moreover, the C-H absorption peaks of benzene extend from 730 to 910 cm^−1^.

### 3.3. Gas Adsorption

**Figure 4:**
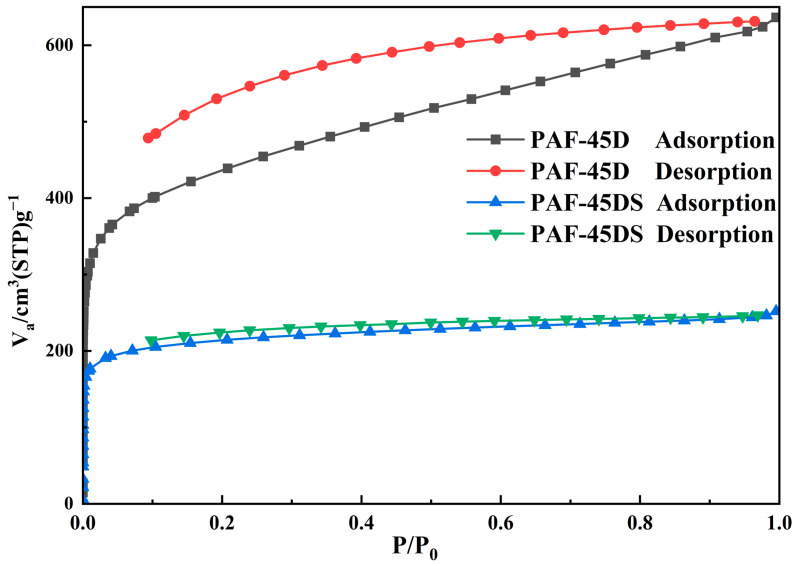
The N_2_ adsorption isotherms of PAF-45D and PAF-45DS.

The N_2_ adsorption isotherm of the activated sample was measured at 77 K to explore the porosity of these PAF materials. All samples were pumped to a vacuum at 423 K for 8 h before the measurement. According to Figure 4, the two materials exhibit typical I type isotherms, indicating substantial and rapid N_2_ uptake at the low-pressure region (P/P_0_ < 0.1). The adsorption and desorption curves of PAF-45D exhibit distinct hysteresis, a phenomenon commonly observed in many PAF networks, attributed to the interaction between nitrogen and the pore channels. After sulfonation, the hysteresis of the adsorption and desorption curves of PAF-45DS is significantly diminished, indicating that the interaction is reduced.

For PAF-45D, the Brunauer–Emmett–Teller (BET) surface area was calculated to be 1571.9 m^2^·g^−1^, and that of PAF-45DS was 806.1 m^2^·g^−1^. The pore size distribution can be obtained by the nonlocal density functional theory (NLDFT) method. The pore sizes of PAF-45D and PAF-45DS are 1.04 nm and 0.44 nm, respectively. These results clearly indicate that the sulfonic acid group occupies the pores of PAF-45D, which reduces the specific surface area and pore size. It also proves the successful introduction of the sulfonic acid group. The water vapor adsorption at 298K was also investigated (Figure S2), demonstrating that the sulfonated material exhibited an enhanced water adsorption capacity due to the presence of hydrophilic sulfonic groups on the frameworks. Despite the reduction in the pore size and surface area of PAF-45DS, it still demonstrates higher water uptake (177 cm^3^/g, STP), surpassing that of PAF-45D (128 cm^3^/g, STP).

### 3.4. Morphological Characteristics

**Figure 5:**
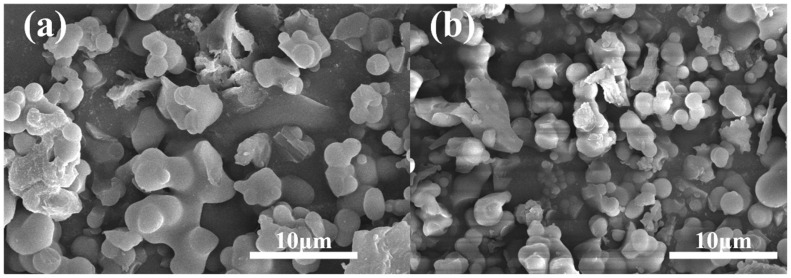
The SEM images of (**a**) PAF-45D and (**b**) PAF-45DS.

The morphological characteristics of the PAF powder and Nafion composite film were obtained by SEM. According to Figure 5, most of the PAF-45D and PAF-45DS particles display irregular spherical shapes, indicating that sulfonation has no significant effect on the morphology of the material. The SEM and EDS images for PAF-45DS were employed to confirm the S elemental content. Figure S3 displays the EDS spectrum obtained from the surface of the sulfonated material. The quantitative analysis results are shown in Table S1. The results indicate the distinct presence of the S element in the PAF-45DS material. The presence of water molecules in the material leads to an excess of oxygen, resulting in an increased O/S ratio.

**Figure 6:**
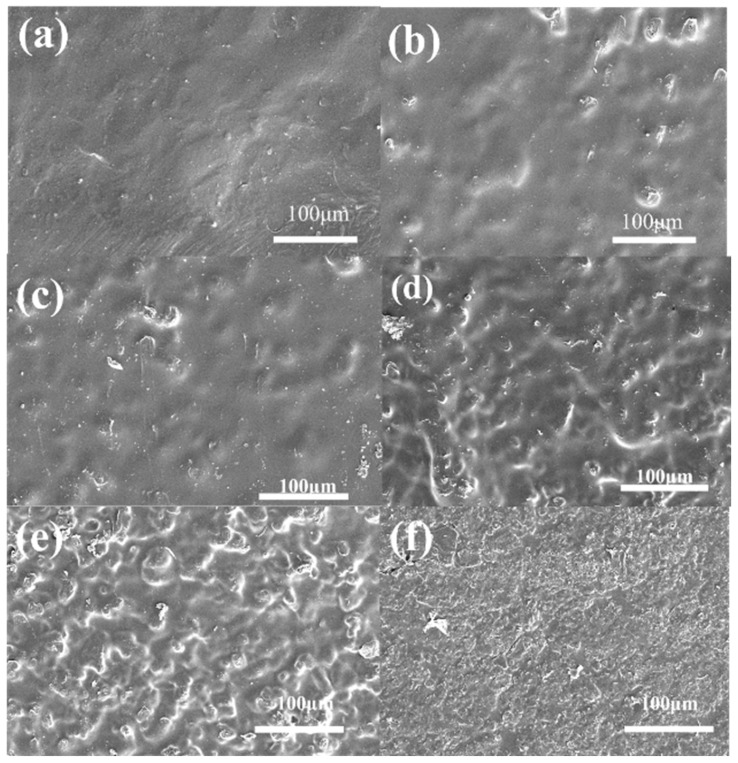
The SEM images of the Nafion composite membranes with different content of PAF-45DS: (**a**) pure Nafion (0 wt.% PAF-45DS) and (**b**–**f**) the content of 2 wt.%, 4 wt.%, 6 wt.%, 8 wt.%, 10 wt.%, respectively.

The morphology of the Nafion composite membranes can be observed in Figure 6. The SEM images captured from the top surfaces of different membranes reveal that as the PAF-45DS mass fraction increases, the solid particles are packed more closely and the composite membranes become progressively rougher. When the quantity increases to 10 wt.%, the Nafion becomes scarcer and crevices can be seen on its surface. This phenomenon is normal when the dispersing phase increases. As the crystal content increases, it may cause the composite membranes to become more brittle and less flexible. An increase in the solid particle content of PAF-45DS in a mixed matrix membrane also results in enhanced brittleness and decreased flexibility.

### 3.5. Proton Conduction of Composite Membranes

**Figure 7:**
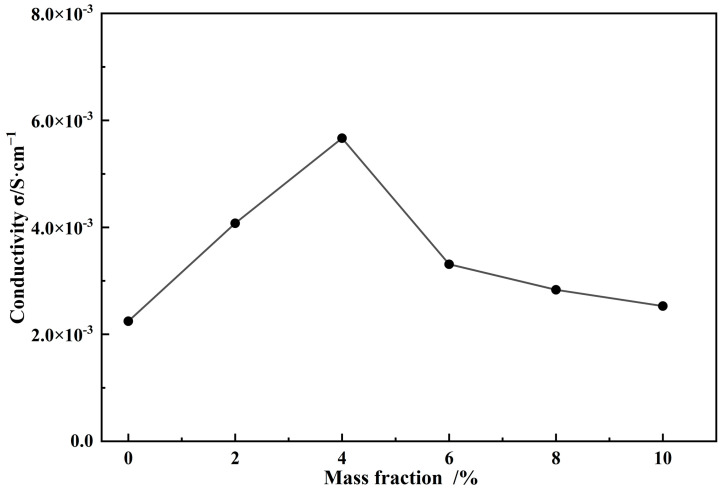
The conductivity of the Nafion composite membranes with different content of PAF-45DS at 80 °C, ~78% RH.

The proton-conductive properties of the Nafion composite membranes were evaluated by using AC impedance spectroscopy at 80 °C and ~78% RH (Figure 7). The detailed Nyquist curves are shown in Figure S4. The proton conductivity of the pure Nafion membrane (0 wt.% PAF-45DS) was 2.245 × 10^−3^ S·cm^−1^ at the same film-forming conditions. The proton conductivity of the composite membranes significantly increased with the addition of PAF-45DS. It can be observed that varying the loading amounts of PAF-45DS from 2 wt.% to 4 wt.% leads to an increase in proton conductivity from 4.077 × 10^−3^ S·cm^−1^ to 5.667 × 10^−3^ S·cm^−1^. These results indicate that the addition of PAF-45DS can enhance the proton conductivity of Nafion membranes at high temperatures.

Under the same film formation conditions, the conductivity of the Nafion film can be increased by nearly 2.5 times. This may be due to the fact that PAF-45DS provides some sulfonic acid groups and its abundant pores can reduce the loss of water molecules at high temperatures. The proton conductivity of the composite membranes reached a peak value at 4 wt.% PAF-45S. Afterwards, as the content increased, the conductivity began to decrease. The conductivity was 3.310 × 10^−3^ S·cm^−1^ (6 wt.%), 2.832 × 10^−3^ S·cm^−1^ (8 wt.%), and 2.528 × 10^−3^ S·cm^−1^ (10 wt.%), respectively. This may be due to the increased doping mass, which reduces the continuity of the Nafion film and leads to a decrease in conductivity. When the temperature was around 80 °C, we observed a decrease in the proton conductivity of the Nafion composite membrane as the temperature increased. Therefore, we were unable to determine the activation energy for proton conduction under the same test conditions.

## 4. Conclusions

In this study, PAF-45D with 1571.9 m^2^·g^−1^ BET surface area was successfully synthesized with AlCl_3_, dichloromethane and biphenyl. After this, PAF-45D was treated by sulfonation, recorded as PAF-45DS. The successful incorporation of sulfonic groups was confirmed through TG, IR, and EDS analyses. Then, a novel Nafion composite membrane was prepared by introducing PAF-45DS. The SEM images show that as the PAF-45DS mass fraction increases, the solid particles are packed more closely and the composite membranes become progressively rougher. The proton conductivity of the Nafion composite membranes increased from 2.245 × 10^−3^ S·cm^−1^ (0 wt.% PAF-45DS) to 5.667 × 10^−3^ S·cm^−1^ (4 wt.%), followed by a gradual decrease to 2.528 × 10^−3^ S·cm^−1^ (10 wt.%). The results show that the conductivity of the Nafion composite membrane can be significantly enhanced by nearly 2.5 times when doping with only a 4% mass fraction of PAF-45DS. To the best of our knowledge, this is an impressive statistic, indicating that the sulfonated PAF-45DS material can improve the proton conductivity of Nafion membranes in high-temperature environments. Such promising materials not only improve the conductivity but also offer a cost-effective solution for advanced composite membranes. They hold great potential for widespread application in the composite film industry.

## Data Availability

Data are contained within the article or Supplementary Materials.

## Supplementary Materials

The following supporting information can be downloaded at https://www.mdpi.com/article/10.3390/polym16152208/s1, Figure S1: The IR spectra of the PAF-45D and PAF-45DS samples; Figure S2: The water uptake of the PAF-45D and PAF-45DS samples at 298 K; Figure S3: SEM image and EDS mapping of PAF-45DS; Table S1: The quantitative analysis results of the test sample; Figure S4: Nyquist curves of PAF-45DS composite films with different doping amounts at ~78%, 80 °C conditions.
