# Supplementary material for: The Incorporation of Sulfonated PAF Enhances the Proton Conductivity of Nafion Membranes at High Temperatures
Source: Polymers (Basel). 2024 Aug 2;16(15):2208. doi: 10.3390/polym16152208 (PMC11314880; doi:10.3390/polym16152208)
Supplement: Supplementary file 1 [file polymers-16-02208-s001.zip › polymers-3040203-supplementary.pdf]

Supporting Information

The incorporation of sulfonated PAF enhances the proton conductivity of Nafion membranes at high temperatures

Kun Cai [1,*], Jinzhu Yu [1], Wenjun Tan [1], Cong Gao [1], Zili Zhao [4], Suxin Yuan [1], Jinghui Cheng [1], Yajie Yang [2,*], and Ye Yuan [3]

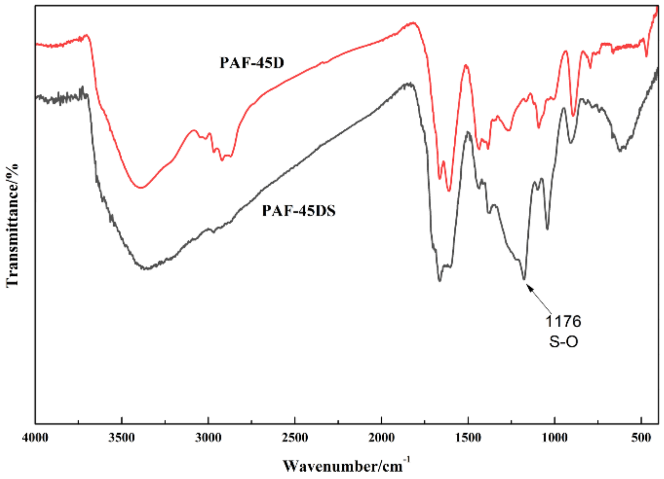

Figure S1 The IR spectra of the PAF-45D and PAF-45DS samples.

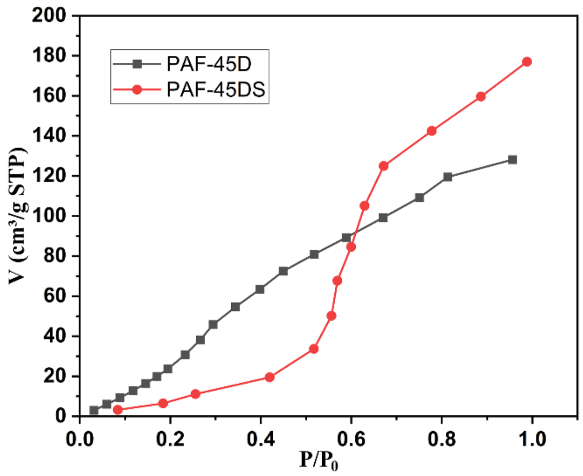

Figure S2 The water uptake of the PAF-45D and PAF-45DS samples at 298K.

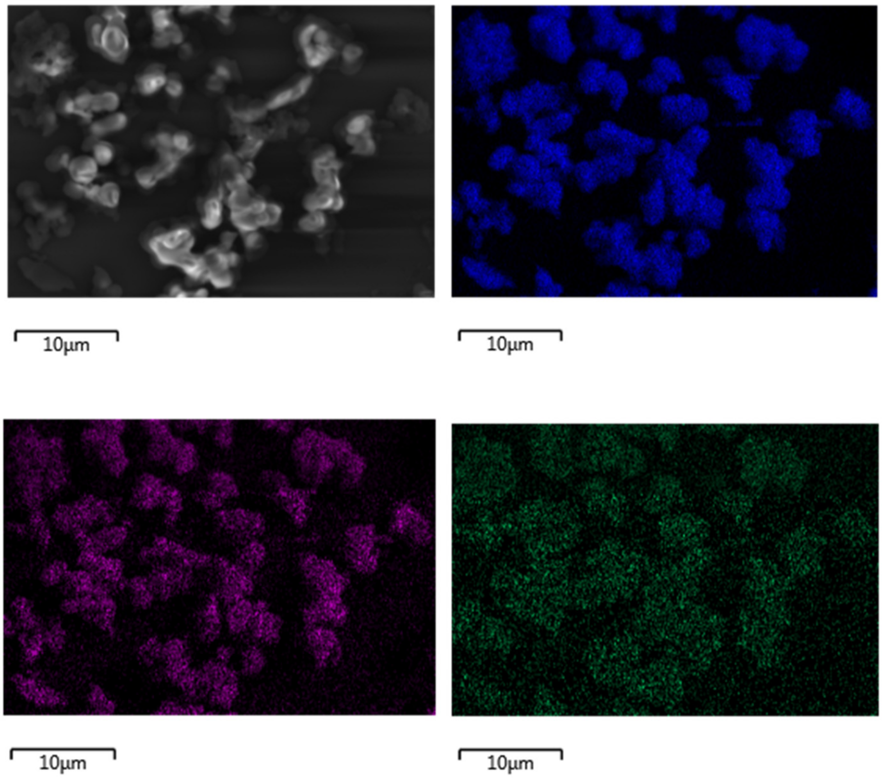

Figure S3 (a) SEM images of PAF-45DS. EDS mappings of C (b), O (c), and S (d) elements.

Table S1 The quantitative analysis results of the test sample.

Element	Line style	Apparent concentration	k rate	wt%	wt% Sigma	Atomic percent
C	K	33.64	0.33639	77.85	0.16	83.52
O	K	11.86	0.03993	18.79	0.15	15.13
S	K	2.49	0.02144	3.36	0.08	1.35
Total				100.00		100.00

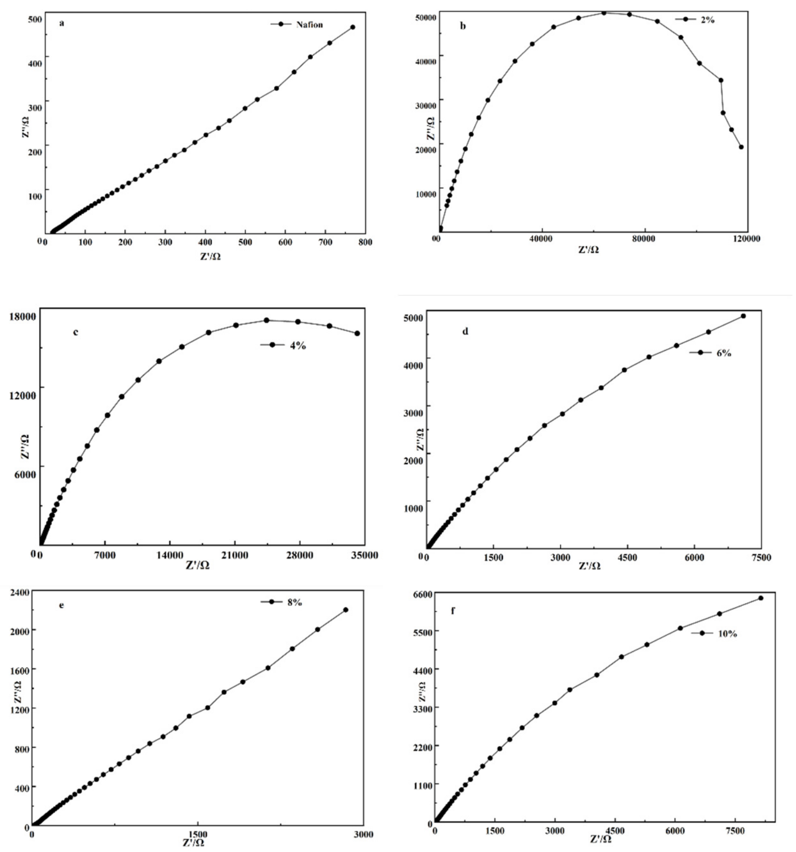

Figure S4 Nyquist curve of PAF-45DS composite films with different doping amounts at ~78%, 80 °C conditions.
